# Enzymatic Degradation of Acrylic Acid-Grafted Poly(butylene succinate-co-terephthalate) Nanocomposites Fabricated Using Heat Pressing and Freeze-Drying Techniques

**DOI:** 10.3390/ma13020376

**Published:** 2020-01-14

**Authors:** Sheng-Hsiang Lin, Hsiang-Ting Wang, Jie-Mao Wang, Tzong-Ming Wu

**Affiliations:** Department of Materials Science and Engineering, National Chung Hsing University, 250 KuoKuang Road, Taichung 402, Taiwan; stanley8977@gmail.com (S.-H.L.); lion1598187@gmail.com (H.-T.W.); D9866023@mail.nchu.edu.tw (J.-M.W.)

**Keywords:** biodegradable, composites, crystallization behavior, degradation, heat pressing technique, freeze-drying technique

## Abstract

Biodegradable acrylic acid-grafted poly(butylene succinate-co-terephthalate) (g-PBST)/organically modified layered zinc phenylphosphonate (m-PPZn) nanocomposites were effectively fabricated containing covalent bonds between the g-PBST and m-PPZn. The results of wide-angle X-ray diffraction and transmission electron microscopy revealed that the morphology of the g-PBST/m-PPZn nanocomposites contained a mixture of partially exfoliated or intercalated conformations. The isothermal crystallization behavior of the nanocomposites showed that the half-time for crystallization of 5 wt % g-PBST/m-PPZn nanocomposites was less than 1 wt % g-PBST/m-PPZn nanocomposites. This finding reveals that increasing the loading of m-PPZn can increase the crystallization rate of nanocomposites. Degradation tests of g-PBST/m-PPZn nanocomposites fabricated using the heat pressing and the freeze-drying process were performed by lipase from *Pseudomonas* sp. The degradation rates of g-PBST-50/m-PPZn nanocomposites were significantly lower than those of g-PBST-70/m-PPZn nanocomposites. The g-PBST-50 degraded more slowly due to the higher quantity of aromatic group and increased stiffness of the polymer backbone. The degradation rate of the freeze-drying specimens contained a more extremely porous conformation compared to those fabricated using the heat pressing process.

## 1. Introduction

Biodegradable polymers, such as poly(1,4-butylene adipate), poly(butylene succinate), poly((butylene succinate)-co-terephthalate) (PBST), poly(butylene succinate-co-adipate) (PBSA), and poly(L-lactic acid), have received significantly attention due to their outstanding biodegradability related to ecological and environmental considerations [1]. Among these biodegradable polymers, aliphatic-aromatic PBST copolymer was synthesized by polycondensation and transesterification using 1,4-butanediol, succinic acid, and dimethylene terephthalate or terephthalic acid [2,3,4,5]. PBST has gained considerable attention because of its excellent mechanical properties exhibited by the aromatic groups in the polymer backbones and advantageous biodegradability of the aliphatic groups [6,7]. PBST has been widely studied compared to another aliphatic polymer, PBSA, because of its enhanced physical properties [8,9].

To further improve these mechanical and thermal properties, adding stiff inorganic material acts as a reinforcing material and nucleating agent in the PBST copolymers as well improving the crystallization behavior [10,11,12]. Wei et al. studied the effects of adding silica and fibrous attapulgite nanoparticles with respect to the crystallization and mechanical properties of nanocomposites [10,11]. The incorporation of inorganic nanoparticles into PBST improved the mechanical properties of nanocomposites as well as accelerated the crystallization rate of PBST. However, only a few investigations have discussed the degradation behavior of PBST copolymers through enzymatic degradation [2,6,7]. To the best of our knowledge, there are no reports discussing the effects of inorganic materials on the degradation and crystallization behavior of PBST copolymers. Consequently, the degradation and crystallization behavior of PBST/inorganic material composites is an important research topic. Recently, two-dimensionally layered zinc phenylphosphonate (PPZn) has received significant interest due to its capability to improve crystallization rates in various polymers [13,14,15]. The biocompatible and nontoxic 1,12-dodecanediamine was utilized to prepare the organically-modified PPZn (o-PPZn) by a co-precipitation method reported previously [16]. A sequence of novel biodegradable nanocomposites was first reported and successfully fabricated using acrylic acid-grafted poly(butylene succinate-co-terephthalate) (g-PBST) and o-PPZn with numerous weight ratios of o-PPZn.

The biocompatible and nontoxic 1,6-diaminohexane, an organic modifier, was used in this study to increase the interlayer distance of PPZn (m-PPZn), and then mixed with g-PBST using identical approach previously developed in our laboratory [16]. The fabricated g-PBST/m-PPZn nanocomposites containing covalent bonds between the g-PBST and m-PPZn are a crucial technique in avoiding the phase separation during the preparation of porous g-PBST nanocomposites by the freeze-drying process. The crystallization behavior and degradation of g-PBST/m-PPZn nanocomposites were studied thoroughly and systematically. To the best of our knowledge, herein, this work is the first report discussing the effects of inorganic materials on the morphology, crystallization behavior, and biodegradability of biocompatible g-PBST/m-PPZn nanocomposites. For the degradation test, two different manufacturing processes were applied to create the changes in surface morphology, namely the heat pressing and the freeze-drying techniques. The effect of surface morphology on the enzymatic degradation was also examined.

## 2. Materials and Methods

### 2.1. Materials

Lipase from *Pseudomonas* sp. (CAS No. 9004-02-8), succinic acid (SA; CAS No. 110-15-6), titanium(IV) isopropoxide (CAS No. 546-68-9), and zinc nitrate (CAS No. 7779-88-6) were obtained from Sigma-Aldrich Chemical Company, Saint Louis, MO, USA. Acrylic acid (AA; CAS No. 79-10-7), azobisisobutyronitrile (CAS No. 78-67-1), 1,4-butanediol (BD; CAS No. 2082-81-7), 1-ethyl-3-(3-dimethylaminopropyl)carbodiimide (EDC; CAS No. 1892-57-5), 1,6-diaminohexane (CAS No. 124-09-4), dimethylene terephthalate (DMT; CAS No. 120-61-6), and tetrabutyl titanate (CAS No. 5593-70-4) were purchased from Alfa Aesar Materials Company, Haverhill, MA, USA. Finally, 1,4-dioxane (CAS No. 123-91-1) was acquired from Macron Fine Chemicals, Radnor, PA, USA. All chemicals were utilized without further purification.

### 2.2. Synthesis of g-PBST/m-PPZn Nanocomposites

Two molar ratios of PBST were synthesized as reported previously [16]. The feed molar ratios of SA to DMT ere 70:30 and 50:50, respectively, with specified amounts of BD, which were assigned as PBST-50 and PBST-70, respectively. The grafting reaction for the fabricated PBST was used a mixture of AIBN and AA at 60 °C for 24 h (henceforth assigned as g-PBST). Since the g-PBST copolymer with the molar ratio of PBT unit was higher than 50%, it could not completely dissolve in the solvent of 1,4-dioxaneused for the sample preparation of the freeze-drying process. The PPZn and 1,6-diaminohexane-modified PPZn (m-PPZn) were synthesized using the approach described in earlier reports [17,18]. Three weight ratios of g-PBST/m-PPZn nanocomposites were prepared containing the covalent linkages between the g-PBST and m-PPZn using the same approach reported previously [16]. Subsequently, g-PBST and its nanocomposites were fully covered by foil, placed between metal flats, and heat pressed with pressing pressure of 142 psi at the temperature approximately 20 °C above their corresponding melting temperatures for 3 min to make the samples for further analyses. For the samples prepared using the freeze-drying process, the fabricated g-PBST/m-PPZn nanocomposites with porous structures were first dissolved in 1,4-dioxane. The prepared solution was poured into a polypropylene test tube. The test tube was slowly moved into a liquid nitrogen bath at a constant rate of 6.8 mm/min to freeze the solution. After totally freezing the solution, the solidified specimen was freeze-dried for 48 h.

### 2.3. Characterization

An X-ray diffractometer (Bruker D8, Karlsruhe, Germany) with Ni-filtered Cu Kα radiation was used for the measurement of wide-angle X-ray diffraction (WAXD). The experiments for the WAXD experiments were performed in the range of 2θ = 1.5°–30° at 1°/min.

Transmission electron microscopy (TEM) was conducted using a JEOL JEM-2010 (Tokyo, Japan). A Reichert Ultracut ultramicrotome was used to prepare the specimens of TEM experiments.

To examine the morphologies of all the test specimens after the degradation, field-emission scanning electron microscopy (FESEM) was performed using a JEOL JSM-6700F (Tokyo, Japan). To avoid charging, the surfaces of samples were coated with gold.

A PerkinElmer Pyris Diamond differential scanning calorimetry (DSC, Waltham, MA, USA) was used to determine the isothermal crystallization behavior, which was calibrated using indium under a nitrogen atmosphere. All specimens were heated to a temperature of about 20 °C above their corresponding melting temperatures (*Tm*^0^) at a scanning rate of 10 °C/min and held for 5 min to remove the thermal history. Consequently, the specimens were quickly cooled to the proposed isothermal crystallization temperatures (*T_cs_*) and held to complete total crystallization. The exothermal curves were illustrated for the analysis. The degree of crystallinity (*X_c_*) was calculated using the enthalpy of fusion (Δ*H_f_*) as reported previously [16].

The weight-average molecular weight (*M_w_*), number-average molecular weight (*M_n_*), and polydispersity PDI = *M_w_/M_n_* of the synthesizedpolymers and composite materials were measured using gel permeation chromatography (GPC; Waters 717plus Autosampler, Waters Instruments, Rochester, NY, USA). Dichloromethane was used as the solvent for the GPC test. The narrow molecular-weight distributions of polystyrene standards were utilized for experimental calibration.

Lipase from *Pseudomonas* sp. with a concentration of 1 mL/mg was used for the degradation test. The test specimens placed in 24-well plates were taken out at 3, 6, 9, 12, and 15 days, washed with distilled water, and vacuum dried. The degradation content was analyzed using the equation *W_weight loss_* (%) = 100((*W*_0_ *− W_t_)/W*_0_), where *W*_0_ is the original weight of a test specimen and *W_t_* is the weight of a test specimen after a selection of degradation times. The average values of three measurements were recorded.

## 3. Results and Discussion

**Figure 1:**
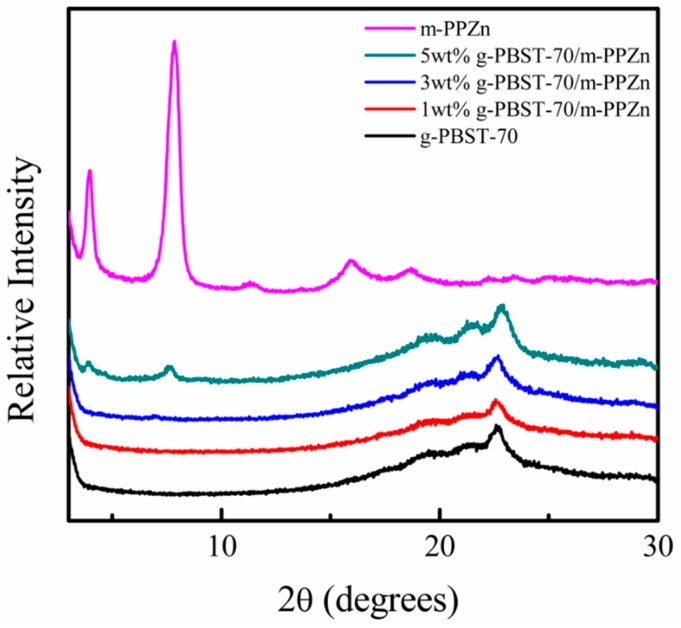
Wide-angle X-ray diffraction (WAXD) patterns of g-PBST-70, m-PPZn, and numerous weight ratios of g-PBST-70/m-PPZn nanocomposites.

**Figure 2:**
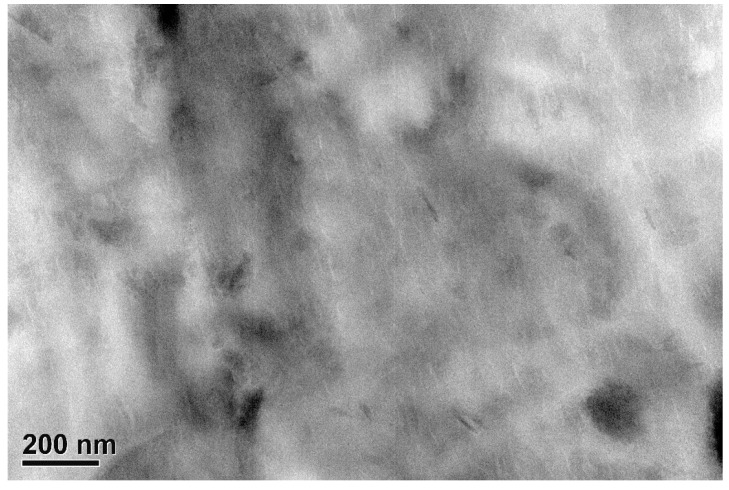
TEM micrographs of 5 wt % g-PBST-70/m-PPZn nanocomposites.

A WAXD technique was used to investigate the structure of g-PBST/m-PPZn nanocomposites. Figure 1 shows the X-ray diffraction profiles of the g-PBST-70/m-PPZn nanocomposites. In order to compare the difference of preparation, the X-ray diffraction curve of m-PPZn is also presented. It is clear that two small diffraction peaks at 2θ = 3.96° and 7.86° were obtained for 5 wt % g-PBST-70/m-PPZn nanocomposites, which were associated with the interlayer spacing of the m-PPZn. The experimental results of the lower loading of m-PPZn showed no clear diffraction peaks observed in this region. The results of WAXD indicated that a mixture of exfoliated and intercalated conformations was found for the g-PBST-70/m-PPZn nanocomposites, while similar experimental data were also observed for the g-PBST-50/m-PPZn nanocomposites. Moreover, a TEM technique was used to directly observe the morphology of g-PBST/m-PPZn nanocomposites. Figure 2 shows the TEM micrographs of 5 wt % g-PBST-70/m-PPZn nanocomposites, where the stacking layers of m-PPZn were partially exfoliated or intercalated inside the g-PBST-70 matrix. The TEM micrographs for 5 wt % g-PBST-50/m-PPZn showed similar morphology. These results were consistent with the WAXD diffraction data.

To understand the effects of crystallization temperature (*T_c_*) on the crystallization behavior of g-PBST/m-PPZn nanocomposites, isothermal crystallization of g-PBST copolyesters with various m-PPZn weight ratios were examined. Isothermal crystallization kinetics were determined using the Avrami equation and illustrated as follows [19,20]:

(1) $$1-X_{t}=\exp(-kt^{n}),$$

where *n* is the Avrami exponent, *X_t_* is the comparative degree of crystallinity at crystallization time *t*, and *k* is the crystallization rate constant. Equation (1) can be conveniently converted into its natural logarithm form as follows:

(2) $$\ln[-\ln(1-X_{t})]=n\ln t+\ln k.$$

The *t*_1/2_ is the time at which the extent of crystallization is completed 50%, which is assigned as the half-time of crystallization and characterized as Equation (3).

(3) $$t_{1/2}={\left(\frac{\ln2}{k}\right)}^{1/n}$$

**Figure 3:**
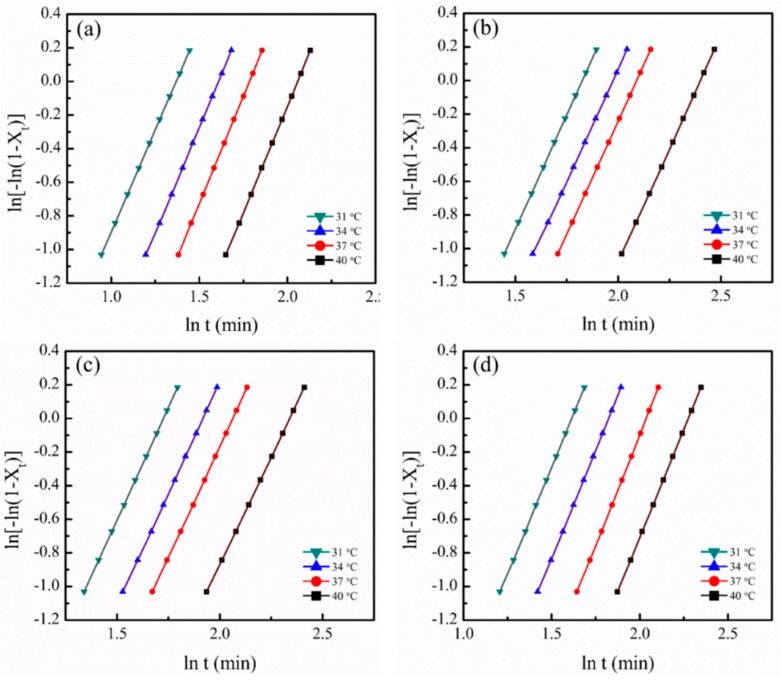
Avrami plots of (**a**) g-PBST-70, (**b**) 1 wt % g-PBST-70/m-PPZn, (**c**) 3 wt % g-PBST-70/m-PPZn, (**d**) 5 wt % g-PBST-70/m-PPZn nanocomposites.

Figure 3 shows the Avrami plots of g-PBST-70 and g-PBST-70/m-PPZn nanocomposites at different *T_cs_*. All profiles were approximately analogous to each other, revealing that the crystallization mechanism of the g-PBST-70 and g-PBST-70/m-PPZn nanocomposites remained the same. Similar findings were also achieved for g-PBST-50/m-PPZn nanocomposites. In order to compare the difference of the samples, the *n*-values, *k*-values, and *t*_1/2_ at various *T_cs_* are shown in Table 1. The value of the Avrami exponent, *n*, represents effective qualifications on the development of crystal growth and the mechanism of nucleation. It was noted that the *n*-values of g-PBST-70 were in the range of 2.43–2.55. The non-integral *n*-values may be a function of mixed crystal growth and the nucleation mechanism [21,22]. Generally, the *n*-values close to 2.5 contribute to an athermal nucleation procedure followed via mixed crystal growth of a two-dimensional and three-dimensional process. The *n*-values of g-PBST-70/m-PPZn nanocomposites were obtained ranging from 2.54 to 2.72, which are similar to g-PBST-70. These findings suggest that the incorporation of m-PPZn into the g-PBST-70 matrix does not alter the mechanism of crystallization for g-PBST-70. Additionally, *t*_1/2_ can be used to investigate the crystallization kinetics of neat g-PBST-70 and g-PBST-70/m-PPZn nanocomposites. For all samples, the values of *t*_1/2_ increased as *T_c_* increased, revealing that the isothermal crystallization rate decreased as the *T_c_* increased. This behavior was attributed to less supercooling at higher *T_c_*. With the addition of 1 wt % m-PPZn into g-PBST-70, the *t*_1/2_ significantly increased from 3.37 to 5.42 min when crystallized at *T_c_* = 31 °C. With the addition of more m-PPZn into the g-PBST-70 copolymers, the *t*_1/2_ slightly decreased. Since the crystalline structure of g-PBST-70 copolymers was determined to be in the crystalline form of aliphatic and flexible PBS, the addition of rigid m-PPZn may have limited the migration and diffusion of g-PBST-70 polymer chains to the packing of PBS crystals, as a result of restricted effects. Therefore, the loading of 1 wt % m-PPZn into g-PBST-70 resulted in an increase of *t*_1/2_. Adding more m-PPZn into the g-PBST-70 serving as a heterogeneous nucleating agent could slightly enhance the crystallization in the nanocomposites. However, the *t*_1/2_ of the g-PBST-70/m-PPZn nanocomposite was higher than that of the neat g-PBST-70 matrix when crystallized at the same *T_c_*. These results suggested that the addition of rigid m-PPZn significantly affected the migration and diffusion of g-PBST-70 polymer chains to the packing of PBS crystals.

Table 1 also illustrates the *n*-values, *k*-values, and *t*_1/2_ at different *T_cs_* for the g-PBST-50/m-PPZn nanocomposites. The *n*-values of g-PBST-50/m-PPZn nanocomposites were in the range of 2.18–2.73. As the crystalline structures of g-PBST-50 copolymers were determined to be in PBT crystalline form with a rigid aromatic unit in the polymer backbone, the incorporation of rigid m-PPZn did not affect the migration and diffusion of g-PBST-50 chains to the arrangement of PBT crystals. The *t*_1/2_ of g-PBST-50/m-PPZn nanocomposites was lower than that of the neat g-PBST-70 matrix when crystallized at the same *T_c_*. By adding more m-PPZn into g-PBST, the *t*_1/2_ decreased with increasing m-PPZn content, implying that m-PPZn accelerated the crystallization of PBT in the nanocomposites. The degree of crystallinity (*X_c_*) was determined using the enthalpy of fusion (Δ*H_f_*) of the DSC heating profile. The values of *X_c_* were 17.4, 16.6, 16.2, and 16.0 for the g-PBST-70, 1 wt % g-PBST-70/m-PPZn, 3 wt % g-PBST-70/m-PPZn, and 5 wt % g-PBST-70/m-PPZn nanocomposites, respectively. A similar tendency was also obtained for the g-PBST-50/m-PPZn nanocomposites.

**Figure 4:**
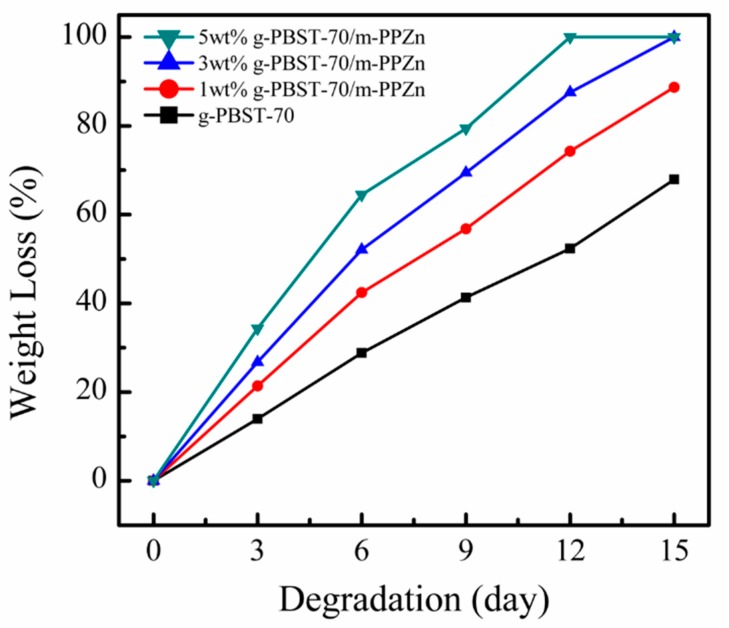
Dependence of the weight loss on the degradation time for the g-PBST-70/m-PPZn nanocomposites prepared using the heat pressing process.

For the degradation test, two different manufacturing processes were applied to create the changes in surface morphology, namely the heat pressing and the freeze-drying techniques. The influences of m-PPZn on the degradation of the g-PBST/m-PPZn nanocomposites were obtained by evaluating the weight loss gravimetrically, determining the change in molecular weight using GPC, and viewing the surface morphology via FESEM [23]. Since m-PPZn cannot be decomposed using lipase, the change in weight loss, molecular weight, and surface morphology after degradation were only related to the g-PBST copolymers. Figure 4 shows the weight losses of the g-PBST-70/m-PPZn nanocomposites fabricated using the heat pressing technique. The time period for the degradation test was dependent on the time necessary for the entire degradation of these test samples. For the g-PBST-70/m-PPZn nanocomposites, the weight loss was about 100% for 5 wt % g-PBST-70/m-PPZn nanocomposites after 12 days, and the weight losses for the neat g-PBST matrix, 1 wt % g-PBST-70/m-PPZn, and 3 wt % g-PBST-70/m-PPZn nanocomposites over the same time period were analyzed to be approximately 52.3%, 74.3%, and 87.6%, respectively. To further compare the degradation behavior, the duration of the degradation test was increased to 15 days. The weight loss tendencies of the g-PBST-50/m-PPZn nanocomposites were comparable to those for the g-PBST-70/m-PPZn nanocomposites. The weight loss values over the various degradation periods for all nanocomposites are summarized in Table 2. It can be clearly seen that the degradation rates of the g-PBST-50 copolymers were extensively lower than those of g-PBST-70 copolymers, which may have contributed to the higher loading of the aromatic group and the increased chain stiffness of the polymer backbone. Meanwhile, the increasing concentrations of m-PPZn also increased the weight loss of samples, indicating that the presence of m-PPZn accelerated the degradation of g-PBST copolymers. This finding could be attributed to the lower *X_c_* for the higher loadings of m-PPZn in the g-PBST matrix.

**Figure 5:**
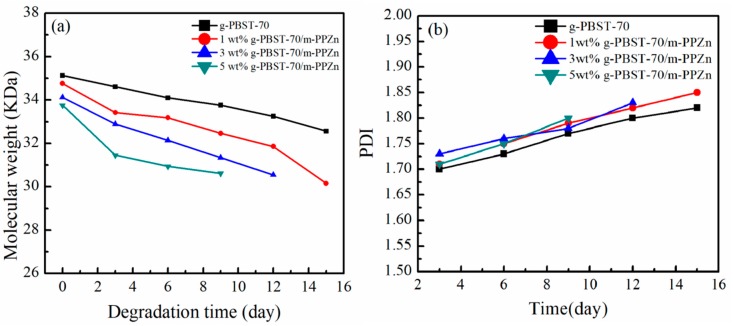
(**a**) Molecular weight and (**b**) polydispersity index (PDI) of residual neat g-PBST-70/m-PPZn nanocomposites after the microbial degradation.

Figure 5 shows the molecular weight (*M_w_*) and polydispersity index (PDI) of g-PBST-70/m-PPZn nanocomposites after degradation testing. It can be seen that the g-PBST-70 copolymer contained the highest *M_w_* of 35 kDa and lowest PDI of 1.68 among the analyzed g-PBST-70/m-PPZn nanocomposites. During the duration of the degradation test, *M_w_* and PDI of all analyzed samples did not significantly change. Similar results were also observed for g-PBST-50/m-PPZn nanocomposites. The *M_ws_* of the g-PBST-50/m-PPZn nanocomposites were about 31 kDa with a PDI of 1.72. According to previous investigations, the *M_w_* and PDI of biodegradable polymers categorized as exo-type hydrolysis activity were almost the same while the number of biodegradable polymers gradually decreased [24,25]. Therefore, our experimental results indicated that the exo-type hydrolysis activity was applied to the degradation behavior of the g-PBST polymer.

**Figure 6:**
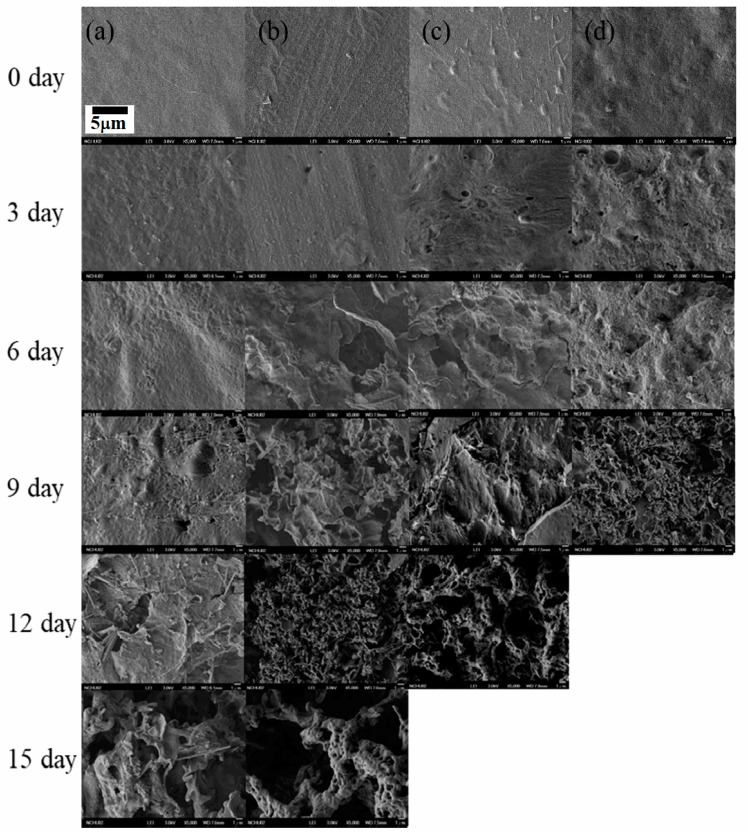
Field-emission scanning electron microscopy (FESEM) images of the microbially degraded (**a**) g-PBST-70, (**b**) 1 wt % g-PBST-70/m-PPZn, (**c**) 3wt % g-PBST-70/m-PPZn nanocomposites, and (**d**) 5wt % g-PBST-70/m-PPZn nanocomposites prepared using the heat pressing process.

The morphologies of the degraded g-PBST-70/m-PPZn nanocomposites investigated via FESEM are presented in Figure 6. Before degradation, the neat g-PBST copolymers showed comparatively smooth surface qualities compared to those of the nanocomposites. Figure 6b illustrates that the roughness of the g-PBST-70 surface increased with the incorporation of 1 wt % m-PPZn. These results also revealed that the addition of m-PPZn enhanced the degradation rate of the g-PBST-70 copolymer. The surface morphologies of more highly loaded m-PPZn into the g-PBST-70 copolymer are shown in Figure 6c,d. From these FESEM micrographs, the degradation rates of 3 wt % and 5 wt % loadings of m-PPZn into g-PBST-70 copolymers were supplementarily enhanced as the loading of m-PPZn increased. After the 15-day degradation test, 3 wt % and 5 wt % g-PBST-70/m-PPZn nanocomposites were fully degraded. Ananalogous tendency was also obtained for the g-PBST-50/m-PPZn nanocomposites.

**Figure 7:**
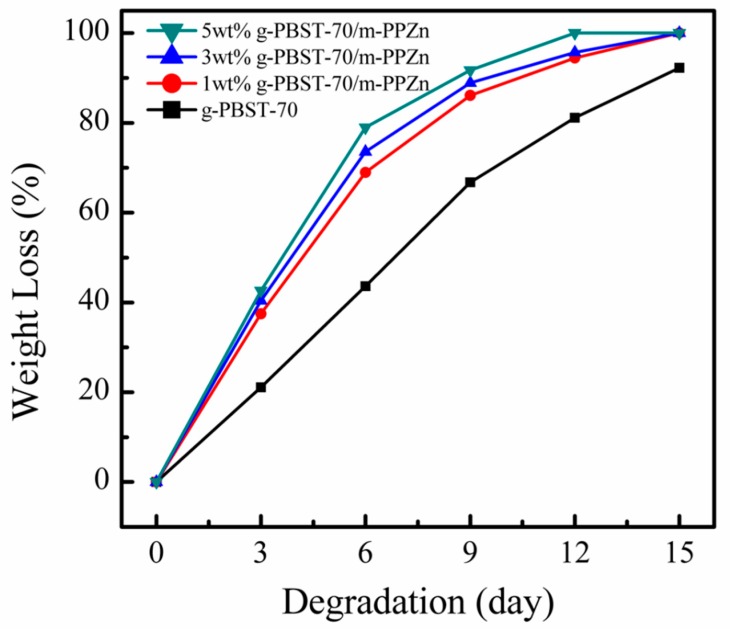
Dependence of the weight loss on the degradation time for the g-PBST-70/m-PPZn nanocomposites prepared using the freeze-drying process.

**Figure 8:**
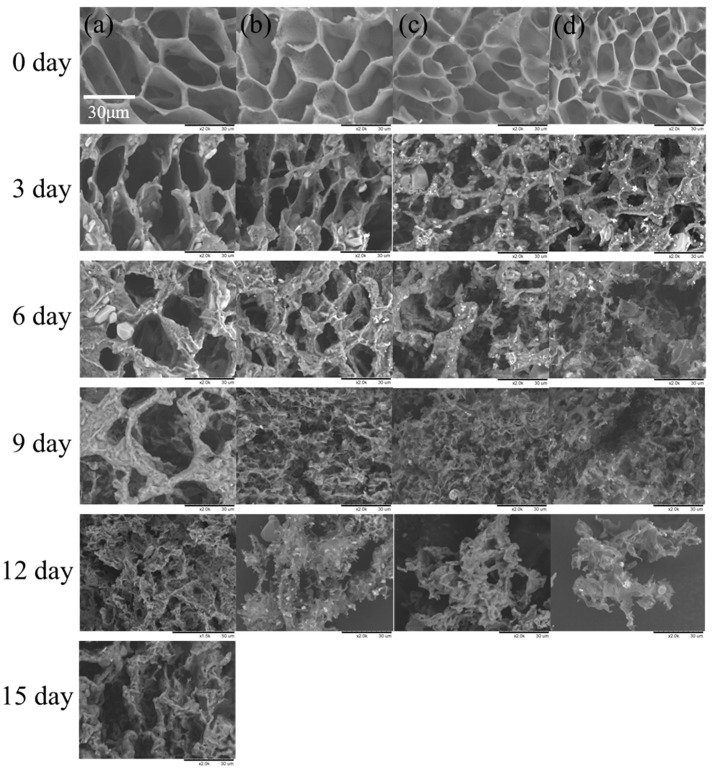
FESEM images of the microbially degraded (**a**) g-PBST-70, (**b**) 1 wt % g-PBST-70/m-PPZn, (**c**) 3 wt % g-PBST-70/m-PPZn nanocomposites, and (**d**) 5 wt % g-PBST-70/m-PPZn nanocomposites prepared using the freeze-drying process.

To investigate the influence of surface morphology on the degradation rates of g-PBST/m-PPZn nanocomposites, a porous structure of the g-PBST/m-PPZn nanocomposites was obtained using the freeze-drying technique. Since the fabricated g-PBST/m-PPZn nanocomposites contained covalent bonds between the g-PBST and m-PPZn, it was a crucial process to avoid phase separation of g-PBST and m-PPZn through the freeze-drying method. Figure 7 shows the weight losses of the g-PBST-70/m-PPZn nanocomposites prepared by means of the freeze-drying method. In comparing the two preparation methods, the time period of degradation test was identical. For the g-PBST-70/m-PPZn nanocomposites, the weight loss was 92.7% for neat g-PBST-70 after a 15-day degradation period, and the weight losses for the g-PBST-70/m-PPZn nanocomposites over the same degradation period as g-PBST-70 were 100%. The values of weight loss for the g-PBST-50/m-PPZn nanocomposites prepared using the freeze-drying method are summarized in Table 3. For the porous sample prepared using the freeze-drying method, the degradation rates of the g-PBST-70/m-PPZn nanocomposites were faster than those of g-PBST-50/m-PPZn nanocomposites. This tendency was the same as the samples prepared using the heat pressing method, but the degradation rates of the samples prepared using the freeze-drying method were much higher than those prepared using the heat pressing method. The molecular weights of the g-PBST-70/m-PPZn and g-PBST-50/m-PPZn nanocomposites remained almost constant while the amount of biodegradable polyesters gradually decreased. The morphologies of the degraded g-PBST-70/m-PPZn nanocomposites investigated via FESEM are presented in Figure 8. Before degradation, the neat g-PBST-70 copolymers and their nanocomposites clearly exhibited different porous sizes. As the loading of m-PPZn increased, the pore sizes were slightly decreased. It is clear that the surface of the g-PBST-70/m-PPZn nanocomposite roughened after the 3-day degradation period, indicating that m-PPZn enhanced the degradation rate of the g-PBST-70 copolymer. The degradation rates of 3 wt % and 5 wt % incorporation of m-PPZn into the g-PBST-70 copolymers were supplementarily enhanced as the loading of m-PPZn increased.

In summary, the degradation rate of biodegradable copolyesters was strongly dependent on the loading of aromatic group and the chain stiffness of the polymer backbone. The incorporation of m-PPZn improved the degradation of the g-PBST copolymers and the degradation rate further increased with increased incorporation of m-PPZn, regardless of the crystalline structure or chemical composition of the g-PBST copolymers. Additionally, the degradation rate of g-PBST containing porous conformations was higher than those of non-porous conformations. From these results, the degradation behavior of the biodegradable polymers is believed to be a surface interaction process as the size of the extracellular enzymes were too large to transport through the interior of the polymer material; hence, they could only affect the surface of the polymer [23]. *t* is believed that the addition of lipase can increase direct contact with the g-PBST polymer chain owing to the chemical characteristic and additional hydrophilic characteristic of m-PPZn, particularly when the ratio of m-PPZn is relatively high and a porous conformation is developed. Similar investigations for biodegradable polymer nanocomposites have been studied previously [23,26,27]. Therefore, the degree of flexible conformation of the succinic acid group and hydrophilic nature of the material with a porous structure are key factors influencing the degradation of g-PBST/m-PPZn nanocomposites.

## 4. Conclusions

A new series of biocompatible and biodegradable g-PBST/m-PPZn nanocomposites were successfully synthesized with covalent bonds between the g-PBST and m-PPZn. Both WAXD and TEM results revealed that the morphology of g-PBST/m-PPZn nanocomposites contained a mixture of intercalated and exfoliated conformations. Using isothermal crystallization kinetics, the *t*_1/2_ for crystallization of the 5 wt % g-PBST/m-PPZn nanocomposites was less than that for the 1 wt % g-PBST/m-PPZn nanocomposites. This result reveals that increased loading of m-PPZn can improve the crystallization rate of nanocomposites. Degradation tests of g-PBST/m-PPZn nanocomposites fabricated using the heat pressing and the freeze-drying process were performed by lipase from *Pseudomonas* sp. The degradation rates of g-PBST-70/m-PPZn nanocomposites were significantly higher than those of g-PBST-50/m-PPZn nanocomposites, which may contribute to the larger number of aromatic groups and increased chain stiffness of the polymer backbone. The degradation rate of freeze-dried specimens with extremely porous conformations was higher than those prepared using the heat pressing method. Therefore, the degree of flexible conformation of the succinic acid group and the hydrophilic nature of a porous material were key factors influencing the degradation of g-PBST/m-PPZn nanocomposites.

## Figures and Tables

**Table 1:** Kinetic parameters of neat g-PBST and g-PBST/m-PPZn nanocomposites isothermally melt crystallized at various *T_c_*.

Sample	*T_c_* (°C)	n	k (min^−n^)	*t*_1/2_ (min)
g-PBST-70	31	2.53	3.59 × 10^−2^	3.37
	34	2.55	1.76 × 10^−2^	4.32
	37	2.51	1.05 × 10^−2^	5.16
	40	2.43	5.39 × 10^−3^	6.78
1 wt % g-PBST-70/m-PPZn	31	2.72	7.00 × 10^−3^	5.42
	34	2.66	5.21 × 10^−3^	6.28
	37	2.69	3.62 × 10^−3^	7.06
	40	2.70	1.53 × 10^−3^	9.63
3 wt % g-PBST-70/m-PPZn	31	2.67	9.90 × 10^−3^	4.90
	34	2.66	6.08 × 10^−3^	5.93
	37	2.64	4.32 × 10^−3^	6.85
	40	2.56	2.50 × 10^−3^	9.00
5 wt % g-PBST-70/m-PPZn	31	2.54	1.66 × 10^−2^	4.35
	34	2.57	9.14 × 10^−3^	5.37
	37	2.63	4.66 × 10^−3^	6.67
	40	2.57	2.88 × 10^−3^	8.44
g-PBST-50	105	2.13	2.15 × 10^−1^	1.73
	110	2.15	6.49 × 10^−2^	2.99
	115	2.00	3.04 × 10^−2^	4.75
	120	2.16	8.10 × 10^−3^	7.78
1 wt % g-PBST-50/m-PPZn	105	2.18	7.55 × 10^−1^	0.96
	110	2.02	3.33 × 10^−1^	1.44
	115	1.85	1.47 × 10^−1^	2.31
	120	1.84	6.82 × 10^−2^	3.51
3 wt % g-PBST-50/m-PPZn	105	2.14	1.15 × 10^0^	0.79
	110	1.95	5.01 × 10^−1^	1.18
	115	1.88	2.30 × 10^−1^	1.80
	120	1.80	1.10 × 10^−1^	2.79
5 wt % g-PBST-50/m-PPZn	105	2.13	1.28 × 10 ^0^	0.75
	110	1.97	5.54 × 10^−1^	1.12
	115	1.82	3.03 × 10^−1^	1.58
	120	1.73	1.43 × 10^−1^	2.50

**Table 2:** Weight loss of the heat pressed g-PBST/m-PPZn nanocomposites measured with various degradation times.

Sample	Weight Loss (%)				
	3 Day	6 Day	9 Day	12 Day	15 Day
g-PBST-70	13.95 ± 0.11	28.81 ± 0.24	41.30 ± 0.38	52.33 ± 0.39	67.90 ± 0.58
1 wt % g-PBST-70/m-PPZn	21.39 ± 0.18	42.43 ± 0.40	56.79 ± 0.51	74.30 ± 0.58	88.71 ± 0.82
3 wt % g-PBST-70/m-PPZn	26.73 ± 0.24	52.12 ± 0.49	69.44 ± 0.74	87.55 ± 0.90	100
5 wt %g-PBST-70/m-PPZn	34.30 ± 0.31	64.47 ± 0.58	79.42 ± 0.79	100	100
g-PBST-50	0.85 ± 0.01	1.85 ± 0.02	2.70 ± 0.03	3.12 ± 0.03	4.16 ± 0.04
1 wt % g-PBST-50/m-PPZn	1.16 ± 0.01	4.38 ± 0.04	6.38 ± 0.08	7.50 ± 0.08	8.16 ± 0.09
3 wt % g-PBST-50/m-PPZn	2.63 ± 0.02	5.68 ± 0.06	7.44 ± 0.09	8.42 ± 0.09	9.90 ± 0.11
5 wt % g-PBST-50/m-PPZn	4.80 ± 0.03	7.96 ± 0.09	9.18 ± 0.10	10.30 ± 0.11	15.53 ± 0.14

**Table 3:** Weight loss of the freeze-dried g-PBST/m-PPZn nanocomposites measured with various degradation times.

Sample	Weight Loss (%)				
	3 Day	6 Day	9 Day	12 Day	15 Day
g-PBST-70	21.11 ± 0.24	43.63 ± 0.48	66.75 ± 0.72	81.13 ± 0.94	92.27 ± 1.17
1 wt % g-PBST-70/m-PPZn	37.50 ± 0.40	68.95 ± 0.76	86.10 ± 0.95	94.44 ± 1.25	100
3 wt % g-PBST-70/m-PPZn	40.42 ± 0.48	73.57 ± 0.82	88.91 ± 1.08	95.72 ± 1.42	100
5 wt %g-PBST-70/m-PPZn	42.61 ± 0.49	78.98 ± 0.89	91.75 ± 1.12	100	100
g-PBST-50	4.89 ± 0.05	7.21 ± 0.08	10.36 ± 0.12	11.88 ± 0.12	14.23 ± 0.16
1 wt % g-PBST-50/m-PPZn	6.67 ± 0.08	9.86 ± 0.11	11.83 ± 0.14	14.57 ± 0.16	16.79 ± 0.18
3 wt % g-PBST-50/m-PPZn	7.22 ± 0.09	11.2 ± 0.14	12.97 ± 0.15	16.19 ± 0.18	18.47 ± 0.20
5 wt % g-PBST-50/m-PPZn	7.98 ± 0.10	12.39 ± 0.16	14.73 ± 0.17	18.33 ± 0.21	20.17 ± 0.22
